# Editorial: Reducing Oral Health Disparities: Social, Environmental and Cultural Factors

**DOI:** 10.3389/fpubh.2017.00298

**Published:** 2017-11-13

**Authors:** Tamanna Tiwari, Sarah Baker, Judith Albino

**Affiliations:** ^1^Department of Community Dentistry and Population Health, University of Colorado Hospital, Aurora, CO, United States; ^2^School of Clinical Dentistry, University of Sheffield, Sheffield, United Kingdom; ^3^Colorado School of Public Health, University of Colorado, Aurora, CO, United States

**Keywords:** oral health inequalities, community engagement, interprofessional health, oral health attitudes, preventive health

**Editorial on the Research Topic**

**Reducing Oral Health Disparities: Social, Environmental and Cultural Factors**

Inequities in oral health are profound worldwide, affecting the overall health and quality of life of millions (1). These inequalities are seen most often in association with racial and ethnic minorities, lower socioeconomic levels, in groups defined by gender or differences in environment and cultural factors, and patterns of utilization of care (2, 3). Identifying and understanding these determinants and their pathways of affecting oral health knowledge, behaviors, care utilization, and ultimately, oral health are the first steps toward finding solutions to these inequalities. To date, biological factors related to oral diseases have received much attention in oral health research, whereas the role of social and cultural determinants in oral disease development and progression has just begun to be recognized (4). This research highlights that interventions designed to reduce disparities should adopt a multilevel approach to identify the modifiable mechanisms and target all determinants of oral health disparities.

The articles included in this Research Topic emphasize the need to understand the determinants of oral health disparities, and they also explore and discuss upstream and community-level strategies to reduce the inequalities that have been created.

In Section I, unique elements of oral health inequalities associated with women and children are discussed. In most cultures, mothers are gatekeepers of the health of the entire family. Wilson et al. reports on associations of maternal attitudes, beliefs, and behaviors with the oral health of children. Wandera and Kasumba extend this topic with discussion of how cultural factors and practices affect the oral health of children in a particular region. They emphasize the importance of reporting such practices in the international community, as a strategy for identifying appropriate approaches for addressing regional inequalities.

Section II of this e-book discusses barriers faced by rural populations, racial minorities, and immigrants in accessing care, suggesting some of the reasons these populations bear the highest disease burdens. Chalmers’ (Chalmers) paper discusses how lack of access to care and insurance for low-income racial minorities drives them to seek care in emergency departments, an approach that is ineffective for patients and creates an unnecessary and high financial burden for the healthcare system. Martin et al. and Minick et al. discuss how lack of insurance and geographic location has been associated with incomplete treatment and difficulties in reimbursement for orthodontic services. Brzoska et al. present similar scenarios for immigrant communities living in Germany. The authors describe several factors, such as age, demographic, social, behavioral, and health-related factors, that are associated with access to preventive dental care for immigrant populations. Doan et al. report that some upstream solutions, such as the expansion of public insurance for adults, have tremendously increased utilization of dental care. Such programs also provide more treatment options for patients and influence the decision-making abilities of patients, moving toward retention of teeth and reduction of extractions.

What promising solutions do we have for the challenge of reducing oral health disparities? Section III presents strategies previously seldom discussed that are beginning to emerge in the educational literature, including intentional efforts to modify and improve dental education to address oral health disparities. Moffat et al. discuss the importance of dental schools and programs in developing future ready practitioners who will be culturally competent and able to adapt to changing circumstances of the community and the world around them. One critical change that was presented by Ramos-Gomez et al. is to move the focus of training from solely surgical treatment to more prevention-oriented training, especially for the pediatric patient population. Ultimately, this approach could be expected to improve the oral health of children through better disease-related risk assessment and targeted prevention, thereby reducing the overall cost of care as well. Cooper et al. have emphasized the role of interprofessional strategies for reducing oral health disparities. Training primary care providers to conduct oral health examinations and preventive interventions, such as fluoride varnish applications and oral health education or counseling, will create an environment of future practitioners in various settings who understand the patterns and severity of oral disease in disparities populations and can help to access care for these individuals. Another approach to addressing disparities is discussed by Cidro et al. and involves engagement of the community decision-making processes related to oral health disparities. Community stakeholders can be involved in designing and implementing activities that will make these programs more acceptable, more welcomed, and more sustainable. Hornsby et al. describe the impact of oral health campaigns on the social environment of communities and motivation of families to make healthier choices.

Disparities persist across the full range of oral health issues and challenges, as reflected by the diversity of topics presented here. In the absence of definitive explanations about the root causes of many of these disparities, we are wise always to assume that some groups will be more affected than others. Similarly, some groups will be more able than others both to understand their challenges and to respond to them, including the challenges associated with accessing services or other resources to meet their health needs. To optimize our ability as caregivers and as researchers who must respond to this complex picture, we need to heighten our sensitivity to differences at all levels. This collection of reports suggests the range of responses that we will want to draw on as we continue to create that greater awareness. For example, individual approaches may be powerful for some and less so for others. Cultural expectations may point to the need for family-oriented or community-level approaches for some groups. We need to remember that one size—or type, or level—of intervention or service rarely will be right for all. A myriad of cultural, sociodemographic, psychosocial, and environmental variables creates a complex array of resources from which we can select the most appropriate information and tools to address specific oral health problems, for specific groups, at specific times and locations. Meanwhile, we must continue to learn from our many colleagues whose collective efforts will continue to add to the array of resources available to all of us.

## Author Contributions

All the authors contributed to the draft of the editorial and have critically reviewed and approved it.

## Conflict of Interest Statement

The authors declare that the research was conducted in the absence of any commercial or financial relationships that could be construed as a potential conflict of interest.
